# Seroprevalence of Viral Enzootic Diseases in Swine Backyard Farms in Serbia

**DOI:** 10.3390/ani13213409

**Published:** 2023-11-03

**Authors:** Vesna Milićević, Dimitrije Glišić, Zorana Zurovac Sapundžić, Bojan Milovanović, Jelena Maletić, Nemanja Jezdimirović, Branislav Kureljušić

**Affiliations:** Institute of Veterinary Medicine of Serbia, Janisa Janulisa 14, 11000 Belgrade, Serbia; dimitrije.glisic@nivs.rs (D.G.); zorana.zurovac@nivs.rs (Z.Z.S.); bojan.milovanovic@nivs.rs (B.M.); jelena.maletic@nivs.rs (J.M.); nemanja.jezdimirovic@nivs.rs (N.J.); branislav.kureljusic@nivs.rs (B.K.)

**Keywords:** enzootic viral disease, backyard farm, PPV, Aujeszky’s disease, PRRS, Swine influenza, Serbia

## Abstract

**Simple Summary:**

Infectious diseases are considered one of the main challenges to pig production despite continuous health management and biosecurity improvement. Hard to control, backyard farms are considered a high-risk infection source for commercial farms. However, as some consumers are concerned about animal welfare in intensive production systems, backyard farms are becoming popular as they respect environmental protection and animal welfare more than commercial farms. Though we discovered that Porcine Parvovirus and Aujeszky’s disease are widely present in backyard pigs from Serbia, almost half are free from the four tested diseases. Being the mirror of wild boar health and the link between wild boars and commercial farms, backyard farms must be controlled as they can interfere with countries’ eradication programs.

**Abstract:**

Contrary to pig farming in developed Western countries, in a large part of the world, pigs are still traditionally kept in small backyard farms, usually for family needs. Their main characteristics are low biosecurity, swill feeding, natural breeding and uncontrolled trade. Given the high number of backyard farms in Serbia and the risk they are thought to pose to intensive pig farming, the main aim of this study was to evaluate the prevalence of major viral diseases of swine among traditionally kept pigs in small holdings with low biosecurity. For this investigation, 222 serum samples from 69 backyard holdings were randomly selected and tested for antibodies to Porcine Reproductive and Respiratory Syndrome virus (PRRSV), Aujeszky’s disease virus (ADV), Porcine Parvovirus (PPV) and Swine influenza Virus (SIV) by enzyme-linked immunosorbent assay (ELISA). The herd-level seroprevalence of PRRS, Aujeszky’s disease and PPV was 2.9%, 27.5% and 37.7%, respectively. Swine influenza seroconversion was not confirmed in any of the tested holdings. Despite widely distributed PPV and AD in backyard farms in Serbia, almost 50% of them are still negative for all the tested diseases. The backyard farms must be monitored, and owners must be educated as their role in eradication programs and obtaining country-free status may be crucial.

## 1. Introduction

The increase in market demands and pork meat consumption was addressed by increasing the number of farming animals and/or improving the efficiency of the pork production sector. However, the larger herd sizes led to the emergence of many enzootic diseases [1]. Although intensive pig farming is leading in utilizing technological and scientific advancements, veterinarians and producers still face transmissible infectious disease outbreaks. Infectious diseases are considered one of the main challenges to pig production despite the continuous health management and biosecurity improvement. The financial impact of direct and indirect losses, such as higher mortality, fewer piglets per sow, less meat produced, lower feed conversion, and healthcare costs, are substantial [2,3]. The viral diseases with the highest economic and health impacts are those causing Porcine Respiratory Disease Complex (PRDC), primarily porcine reproductive and respiratory syndrome (PRRS), porcine circovirus type 2 infection (PCV-2), and swine influenza (SIV). Aujeszky’s disease (AD) and porcine parvovirus (PPV) infection are also contagious diseases with significant economic impact. However, AD has been eradicated in many European countries, the U.S., New Zealand and Japan [4], and PPV infection is controlled by vaccination [5].

Contrary to pig farming in developed western countries, in many parts of the world, pigs are still traditionally kept in small backyard farms, usually for family needs. These farms are invisible and uncontrolled by state veterinarians. Their main characteristics are low biosecurity, swill feeding, natural breeding and uncontrolled trade. Thus, since the health status of backyard farms is unknown, they are considered a high-risk infection source for commercial farms [6,7]. Furthermore, the country’s disease status can be compromised since disease control is hardly achievable. As some consumers are concerned about animal welfare in intensive production systems, backyard farms are becoming popular nowadays as they respect environmental protection and animal welfare more than commercial farms, which are viewed as socially unacceptable [8]. In Serbia, most pig farms are small, backyard farms [9], contrary to the EU where only 0.7% of pigs are kept outdoors [10]. Given the high number of backyard farms in Serbia and the risk they are thought to pose to intensive pig farming, the main aim of this study was to evaluate the prevalence of major viral diseases of swine among traditionally kept pigs in small holdings with low biosecurity. PRRS, AD, PPV and SIV were selected based on their prevalence and significance for commercial pig farming in Serbia, where AD is still present, and no eradication program exists. Considering how the pigs in backyards are raised, the working hypothesis was that the prevalence of selected viral diseases is more likely similar to those in the wild boar population rather than on commercial farms.

## 2. Materials and Methods

For this investigation, 222 serum samples from 69 backyard holdings were randomly selected from the classical swine fever (CSF) surveillance conducted from March to August 2023. Each pig residing on the selected holdings was sampled and tested. The data on the holding category according to the total number of swine and the number of sows were obtained from the State Central database. For the antibody detection, commercial ELISA kits were used following manufacturer’s instructions: PrioCHECK PRRSV Ab, Prionics, INgezim Influenza A, Gold Standard Diagnostics, PrioCHECK PRV gB ELISA Prionics and INgezim PPV Compac, and Gold Standard Diagnostics.

Test results are used to classify sera as seropositive or seronegative based on manufacturer-defined breakpoints. The chi-square test was used to evaluate the significance level of association between farm categories and disease seroprevalence.

## 3. Results

According to the total number of swine on a farm, all selected holdings comprised up to 10 pigs, with an average of 3 pigs per farm. According to the number of sows, three farms (4.3%) had no sows, 46 farms (66.7%) kept up to two sows, and 20 farms (29%) belonged to the category with 3–10 sows (Table 1).

**Figure 1:**
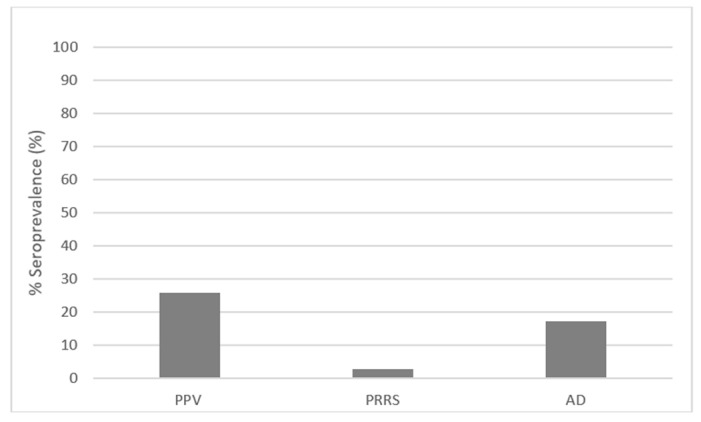
Overall seroprevalence of PRRS, AD and PPV in backyard farms in Serbia.

Out of 222 samples, PPV antibodies were detected in 57 animals, AD antibodies in 38 and PRRS seropositivity in 6 animals. The overall seroprevalence was 2.7% for PRRS, 17.1% for AD and 25.7% for PPV (Table 2, Figure 1). The individual results are given in the Supplementary Material.

The herd-level seroprevalence of PRRS, Aujeszky’s disease and PPV was 2.9%, 27.5% and 37.7%, respectively (Table 1). SIV seroconversion was not confirmed in any of the tested holdings. The proportion of seronegative backyard herds for the four diseases was 49.3%. On two farms (2.9%), only PRRS antibodies were detected. PPV and AD seropositivity were proved in 20.3% and 10.1% of backyard farms, respectively. Simultaneous PPV and Aujeszky’s disease antibodies were detected in 17.4% of farms (Table 3).

Within a herd, the seroprevalence ranged from 20 to 100% for PPV and 33 to 100% for Aujeszky’s disease. PRRS within-herd seroprevalence was 100% in both holdings where seroconversion was confirmed. A chi-square test showed no significant association between farm categories and seroprevalence (the chi-square statistic is 3.881; the *p*-value is 0.422348; the result is insignificant at *p* < 0.05).

## 4. Discussion

Outdoor farming allowed in the EU can be considered different from traditionally kept pigs in backyards in the Balkans. While outdoor farming in the EU means the permanent or temporary keeping of pigs outdoors, the pigs in backyard farms in the Balkans are usually kept closed with no outdoor access. This practice is seen in other countries, such as Cyprus [10]. However, wild boars and domestic pigs may come into contact in certain parts of Serbia, leading to the possibility of boar–pig hybrids.

In the context of biosecurity measures, farms in Serbia are classified into four distinct categories. Among these, family farm type B and backyard farms, which were the focus of this study, are characterized by minimal to nonexistent biosecurity protocols [11]. Further, pig farms in Serbia are divided according to the number of pigs (up to 10 animals, 11–50 animals, 51–100 animals, 101–500 animals, and more than 500 animals) and the number of sows (no sows, 1–2 sows, 3–10 sows, 11–50 sows, and more than 50 sows). This study was conducted on backyard farms with up to 10 animals, farms with no sows and farms keeping 1–2 and 3–10 sows.

Backyard farms in Serbia are distributed throughout the country. However, they are most common in the western Mačvanski and Sremski regions and central Serbia. Many backyard farms in the East house domestic pigs, which roam freely in the forests during the day in search of food [12].

A typical backyard farm in Serbia is partially fenced or unfenced, with no control over persons visiting the animals or quarantining newly purchased animals [11]. However, the interaction between wild and domestic pigs and potential hybridization presents a significant risk for introducing various pathogens or novel strains into the backyard pig population, with potential implications for biosecurity and disease control. Another concerning fact is that it enabled the contact of pigs on the holdings with other animal species, wild boars, among others. Thus, pathogen transmission is uncontrollable and almost certain. Although forbidden, swill feeding is still a widely distributed practice. As stated before, natural breeding, uncontrolled trade and the unknown health status of residing animals are additional concerns to discuss regarding threatening disease spread. Backyard farms, thus, co-exist independently with industrial farming. At the same time, humans are the main link between them, as backyard farmers are usually employed at commercial farms. While regulations may prohibit the keeping of pigs in domestic or non-commercial environments, enforcing these rules can be challenging, leading to potential non-compliance. Therefore, backyards are considered the main risk of pathogen introduction into a commercial farm [13].

This study revealed that SIV is likely not present in backyard pigs, while PRRS seroprevalence is very low (2.7%). Conversely, PPV and AD are commonly present in the backyard swine population in Serbia. The initial hypothesis is confirmed, knowing the epidemiology of selected diseases and their distribution in wild boar [14,15].

SIV and PRRS are typical for intensive production since they can be efficiently transmitted via different routes. They require dense populations and naïve animals that are undoubtedly present in different production phases [16]. Conversely, as none of these conditions are fulfilled in backyard farms, the more common diseases are those transmissible from wild boar, including parasites [16]. Furthermore, owners can be considered critical mechanical vectors and, even more, an infection source of SIV for pigs kept in backyard settings. With poultry commonly freely bred in backyards and making contact with pigs, influenza virus reassortments should also be anticipated [17].

SIV in Serbian commercial farms is the most common respiratory disease [18], represented by H1N1, including the H1N1pdm09 lineage and H3N2 subtypes [19]. The seroconversion in the wild boar population in Serbia has not been reported [14]. In the wave of influenza outbreaks across Europe in recent years, Serbia experienced specific incidences of the disease within backyard poultry populations. During the 2016/2017 period, a total of four outbreaks were officially recorded, followed by a subsequent occurrence of three outbreaks in the 2021/2022 period [20]. Despite the common practice of keeping pigs and poultry together in backyards, no spillover and seroconversion in backyard pigs in this study were detected, which might be due to the short life of backyard pigs and fast actions to limit the infection in poultry [20]. A similar situation with sporadic spillovers from poultry to pigs is seen in Europe [21]. However, the time when the sampling was performed did not overlap with the typical influenza season in fall and winter, and the short life of anti-influenza virus antibodies could also contribute to the negative results.

PRRS, the most significant disease in commercial farming, is also common in Serbian farms, with seroprevalence of up to 70% depending on the region [22]. However, according to this study’s results, the prevalence depends on the farm type, as the backyard farms are commonly free from PRRS. For this reason, it was decided to eradicate PRRS from backyard farms in Hungary [23]. Our findings regarding PRRS are consistent with generally low PRRS seroprevalence in wild boar [24,25]. Backyard population and wild boars are not reservoirs of PRRS, as the prevailing conditions do not provide efficient virus transmission and maintenance.

PPV is enzootic in wild boar and domestic pigs [26]. PPV is widely distributed in European wild boar populations, with the seroprevalence reaching 100% [27]. In Serbia, 37.7% of backyard farms are seropositive for PPV, whereas the average seroprevalence within the farm is 65%. Similar results are reported from other countries with most backyard farms [28,29]. Except for reproductive failure in gilts, the infection is inapparent in domestic pigs and wild boar [29]. Due to the virus resistance, contaminated facilities and equipment are the primary source of the virus in backyard farms, which is why the animals become infected and develop an immune response very early, which can explain the high within-herd prevalence.

While members of the Suidae family are the natural hosts of the Pseudorabies virus causing Aujeszky’s disease, wild boars are considered to be its reservoir. In industrial pig farming, AD causes substantial losses. Thus, it is one of the primary diseases for eradication. However, in wild boar and feral pigs, AD is often subclinical and with unspecific clinical signs. In Serbia, there is no control or eradication plan for AD in domestic pigs. The indirect indicator of AD prevalence in domestic pigs, primarily in backyards, are the outbreaks in carnivores, mainly diagnosed within the rabies surveillance program regarding differential diagnosis (unpublished data).

An AD and PPV coinfection was the second most common finding in backyard farms in Serbia. Concurrent infections in intensive pig farms are also common. The most frequently reported is AD, which weakens the pig’s immunity and enables other infections such as PCV2, CSF and PRRS. Thus, given the latency of AD and resistance and infective pressure of PPV, the coinfections are not surprising findings in our backyard farms [30].

Biosecurity and owner awareness are vital in preventing pigs from being exposed to pathogens through contact with wild boar. A strong correlation has been reported between hepatitis E seroprevalence in wild boar and close-contact domestic pigs. Thus, the seroprevalence in domestic pigs is significantly lower due to limited exposure. On the contrary, the virus circulation and transmission between domestic and wild boar depend on the disease type and cannot be considered as a rule. AD has been shown to have independent cycles in these populations [31].

This study reveals that one-quarter of backyard pig farms are seropositive to AD, whereas the within-herd prevalence is sometimes even 100%. Similarly, the average seroprevalence in wild boar populations in Europe is around 30% [32,33], but can reach even 100% in certain subpopulations [34].

Undoubtedly, the direction of disease transmission is mainly from backyards to commercial farms. The other direction certainly can happen, but this is hardly probable due to the specific conditions in backyard settings.

## 5. Conclusions

Despite widely distributed PPV and AD in backyard farms in Serbia, almost 50% of them are still negative for all the tested diseases. Thus, monitoring backyard farms’ health status must be contemplated, emphasizing the need to improve biosecurity measures. The education of owners and their role in eradication programs and obtaining country-free status may be crucial.

## Figures and Tables

**Table 1:** Herd seroprevalence according to the farm category.

Farm Category	PRRS			PPV			AD		
	No. of Seronegative Herds	No. of Seropositive Herds	Seropositive Herds (%)	No. of Seronegative Herds	No. of Seropositive Herds	Seropositive Herds (%)	No. of Seronegative Herds	No. of Seropositive Herds	Seropositive Herds (%)
No sows	3	0	0.00	2	1	33.33	2	1	33.33
1–2 sows	45	1	2.17	29	17	36.96	35	11	23.91
3–10 sows	19	1	5.00	12	8	40.00	13	7	35.00
TOTAL	67	2	2.9	43	26	37.7	50	19	27.5

**Table 2:** Proportion of seropositive animals on the farms regarding the number of sows.

Farm Category	PRRS			PPV			AD		
	No. of Seronegative Animals	No. of Seropositive Animals	Seropositive Animals (%)	No. of Seronegative Animals	No. of Seropositive Animals	Seropositive Animals (%)	No. of Seronegative Animals	No. of Seropositive Animals	Seropositive Animals (%)
No sows	0	6	0.00	2.00	4.00	33.33	1.00	5.00	16.67
1–2 sows	2	115	1.71	32.00	85.00	27.35	14.00	103.00	11.97
3–10 sows	4	95	4.04	23.00	76.00	23.23	23.00	76.00	23.23
TOTAL	6	216	2.7	57.00	165.00	25.7	38.00	184.00	17.1

**Table 3:** The seropositivity concerning farm category by sow numbers.

	No. of Herds with No Sows (%)	No. of Herds with 1–2 Sows (%)	No. of Herds with 3–10 Sows (%)	Total No. of Herds (%)
Seronegative farm	2 (66.7)	24 (52.2)	8 (40.0)	34 (49.3)
PRRS seropositive farm	0 (0.0)	1 (2.2)	1 (5.0)	2 (2.9)
PPV seropositive farm	0 (0.)	10 (21.7)	4 (20.0)	14 (20.3)
AD seropositive farm	0 (0.00)	4 (8.7)	3 (15.0)	7 (10.1)
PPV + AD seropositive farm	1 (33.3)	7 (15.2)	4 (20.0)	12 (17.4)

## Data Availability

Data available on request from the authors.

## Supplementary Materials

The following supporting information can be downloaded at: https://www.mdpi.com/article/10.3390/ani13213409/s1, Table S1: Individual results with data on the number of pigs and type of farm
